# Supplementary material for: Noticeable Quantities of Functional Compounds and Antioxidant Activities Remain after Cooking of Colored Fleshed Potatoes Native from Southern Chile
Source: Molecules. 2021 Jan 9;26(2):314. doi: 10.3390/molecules26020314 (PMC7827549; doi:10.3390/molecules26020314)
Supplement: Supplementary file 1 [file molecules-26-00314-s001.pdf]

Figure S1: Mass spectrometry (MS/MS) spectra under positive ionization of anthocyanins from *Solanum tuberosum*. Identifications of signals according Table 1 and Figure 1.

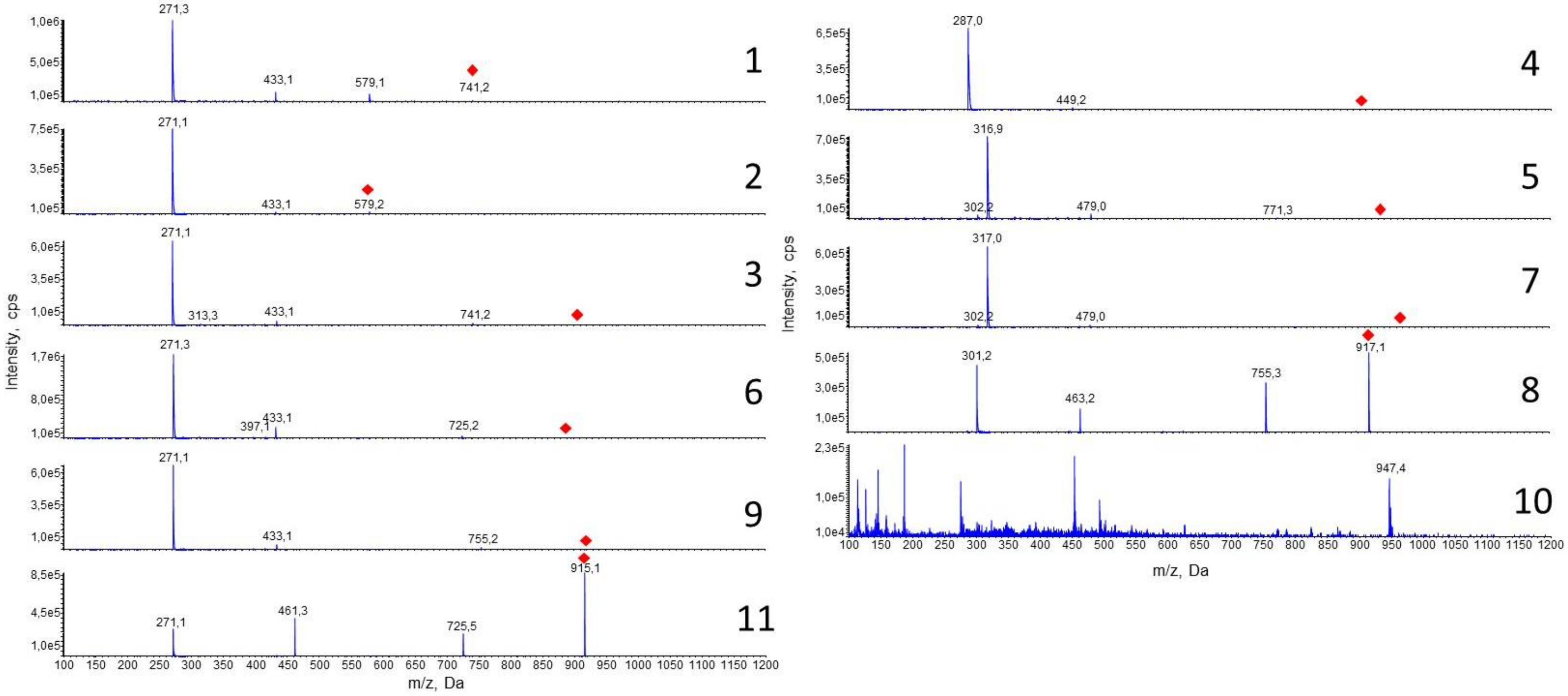

Table S1: Individual anthocyanin concentrations (g kg^{-1} fresh weight) by HPLC-DAD in fresh samples of *Solanum tuberosum*. Identifications according Table 1 and Figure 1. Different letters in a column indicate the presence of statistically significant differences according the Tukey's multiple range test (P ≤ 0.05; n = 3). Where nd: not detected.

Genotype	Peak 1	Peak 2	Peak 3	Peak 4	Peak 5	Peak 6	Peak 7	Peak 8	Peak 9	Peak 10	Peak 11
CB2011.104	nd	nd	nd	0.146 ± 0.000 a	0.903 ± 0.001 a	nd	nd	0.048 ± 0.000 hi	nd	0.033 ± 0.000 e	nd
CB2011.119	nd	nd	nd	0.029 ± 0.000 c	0.349 ± 0.002 e	0.036 ± 0.000 h	nd	0.160 ± 0.000 b	nd	0.029 ± 0.000 e	nd
CB2011.189	nd	nd	nd	0.010 ± 0.000 k	0.080 ± 0.000 o	0.009 ± 0.000 m	nd	0.027 ± 0.000 m	nd	nd	nd
CB2011.247	nd	nd	nd	0.013 ± 0.000 j	0.103 ± 0.000 n	0.040 ± 0.000 fg	nd	0.084 ± 0.000 f	nd	nd	nd
CB2011.569	nd	nd	nd	0.014 ± 0.000 i	0.121 ± 0.002 m	0.008 ± 0.000 m	nd	0.051 ± 0.001 h	nd	0.155 ± 0.003 b	nd
CB2011.616	nd	nd	nd	0.011 ± 0.000 k	0.105 ± 0.002 n	0.016 ± 0.000 jk	nd	0.043 ± 0.001 j	nd	0.004 ± 0.000 ghi	nd
CB2012.096	nd	nd	nd	0.024 ± 0.001 e	0.261 ± 0.002 h	0.018 ± 0.000 j	nd	0.068 ± 0.000 g	nd	0.012 ± 0.000 fg	nd
CB2012.196	nd	0.004 ± 0.000 b	nd	0.022 ± 0.000 f	0.369 ± 0.001 d	nd	nd	nd	nd	nd	nd
CB2011.211	nd	nd	nd	0.020 ± 0.000 g	0.208 ± 0.001 k	0.034 ± 0.000 i	nd	0.090 ± 0.000 e	nd	0.009 ± 0.000 fgh	nd
Pirata	nd	nd	nd	0.012 ± 0.000 j	0.080 ± 0.002 o	0.012 ± 0.000 l	nd	0.030 ± 0.001 l	nd	0.004 ± 0.000 ghi	nd
CB2011.280	nd	nd	nd	0.025 ± 0.000 d	0,318 ± 0.006 g	0.046 ± 0.000 e	nd	0.107 ± 0.002 d	nd	nd	nd
CB2011.273	nd	nd	nd	0.017 ± 0.000 h	0.214 ± 0.006 j	0.042 ± 0.001 f	nd	0.121 ± 0.003 c	nd	nd	nd
CB2011.098	nd	nd	nd	0.057 ± 0.000 b	0.343 ± 0.001 f	0.059 ± 0.000 d	0.071 ± 0.000 a	0.160 ± 0.001 b	nd	0.047 ± 0.001 d	nd
CB2011.253	nd	nd	nd	0.014 ± 0.000 i	0.205 ± 0.000 k	0.011 ± 0.000 g	0.009 ± 0.000 d	0.038 ± 0.000 e	nd	0.015 ± 0.000 f	nd
CB2012.076	nd	nd	nd	0.003 ± 0.000 l	0.019 ± 0.000 s	0.003 ± 0.000 n	0.004 ± 0.000 f	0.005 ± 0.000 n	nd	0.002 ± 0.002 hi	nd
CB2012.176	nd	nd	nd	Nd	0.237 ± 0.000 i	nd	nd	nd	nd	nd	nd
CB2012.347	nd	nd	nd	Nd	nd	nd	nd	nd	nd	nd	nd
CB2011.122	nd	nd	nd	Nd	0.145 ± 0.001 l	0.015 ± 0.000 k	0.011 ± 0.000 c	0.047 ± 0.000 i	nd	nd	nd
CB2011.375	nd	nd	nd	Nd	0.050 ± 0.000 q	0.009 ± 0.000 m	nd	0.027 ± 0.000 m	nd	nd	nd
CB2012.028	nd	nd	nd	Nd	0.021 ± 0.000 s	0.003 ± 0.000 n	0.003 ± 0.000 g	0.005 ± 0.000 n	nd	nd	nd
CB2012.063	nd	nd	nd	Nd	0.034 ± 0.000 r	0.004 ± 0.000 n	0.004 ± 0.000 e	0.007 ± 0.000 n	nd	nd	nd
CB2012.128	nd	nd	nd	Nd	0.007 ± 0.000 t	nd	nd	nd	nd	nd	nd
CB2012.208	nd	nd	nd	Nd	0.665 ± 0.001 b	0.058 ± 0.000 d	nd	0.179 ± 0.000 a	nd	nd	nd
CB2012.253	nd	nd	nd	Nd	0.584 ± 0.000 b	0.039 ± 0.000 l	nd	0.090 ± 0.000 k	nd	nd	nd

CB2012.350	nd	nd	nd	Nd	0.006 ± 0.000 t	nd	nd	nd	nd	nd	nd
CB2012.361	nd	nd	nd	Nd	0.019 ± 0.001 s	0.147 ± 0.004 b	nd	nd	0.156 ± 0.004 a	0.353 ± 0.014 a	nd
CR2012.363	nd	nd	nd	Nd	nd	nd	nd	nd	0.009 ± 0.000 c	nd	nd
CR2002.8	nd	nd	0.031 ± 0.000 a	Nd	nd	nd	nd	0.170 ± 0.000	nd	nd	0.018 ± 0.000 b
TR2012.078	0.057 ± 0,000 a	0.015 ± 0.000 a	nd	Nd	nd	0.170 ± 0.000 a	0.023 ± 0.001 b	nd	nd	nd	0.022 ± 0.000 a
TY2012.365	nd	nd	nd	0.002 ± 0.000 m	0.003 ± 0.000 tu	0.017 ± 0.000 jk	nd	nd	0.015 ± 0.000 b	0.033 ± 0.000 e	nd
CB2011.568	nd	nd	nd	Nd	nd	0.074 ± 0.000 c	nd	nd	nd	0.107 ± 0.000 c	nd
BWF	nd	nd	nd	Nd	nd	nd	nd	nd	nd	nd	nd
VR808	nd	nd	nd	Nd	nd	nd	nd	nd	nd	nd	nd

Table S2: Individual anthocyanin concentrations (g kg^{-1} fresh weight) by HPLC-DAD in cooked samples of *Solanum tuberosum*. Identifications according Table 1 and Figure 1. Different letters in a column indicate the presence of statistically significant differences according the Tukey's multiple range test ($P \leq 0.05$; $n = 3$). Where nd: not detected.

Genotype	Peak 1	Peak 2	Peak 3	Peak 4	Peak 5	Peak 6	Peak 7	Peak 8	Peak 9	Peak 10	Peak 11
CB2011.104	nd	nd	nd	0.146 ± 0.000 a	0.903 ± 0.001 a	nd	nd	0.048 ± 0.000 hi	nd	0.033 ± 0.000 e	nd
CB2011.119	nd	nd	nd	0.029 ± 0.000 c	0.349 ± 0.002 e	0.036 ± 0.000 h	nd	0.160 ± 0.000 b	nd	0.029 ± 0.000 e	nd
CB2011.189	nd	nd	nd	0.010 ± 0.000 k	0.080 ± 0.000 o	0.009 ± 0.000 m	nd	0.027 ± 0.000 m	nd	nd	nd
CB2011.247	nd	nd	nd	0.013 ± 0.000 j	0.103 ± 0.000 n	0.040 ± 0.000 fg	nd	0.084 ± 0.000 f	nd	nd	nd
CB2011.569	nd	nd	nd	0.014 ± 0.000 i	0.121 ± 0.002 m	0.008 ± 0.000 m	nd	0.051 ± 0.001 h	nd	0.155 ± 0.003 b	nd
CB2011.616	nd	nd	nd	0.011 ± 0.000 k	0.105 ± 0.002 n	0.016 ± 0.000 jk	nd	0.043 ± 0.001 j	nd	0.004 ± 0.000 ghi	nd
CB2012.096	nd	nd	nd	0.024 ± 0.001 e	0.261 ± 0.002 h	0.018 ± 0.000 j	nd	0.068 ± 0.000 g	nd	0.012 ± 0.000 fg	nd
CB2012.196	nd	0.004 ± 0.000 b	nd	0.022 ± 0.000 f	0.369 ± 0.001 d	nd	nd	nd	nd	nd	nd
CB2011.211	nd	nd	nd	0.020 ± 0.000 g	0.208 ± 0.001 k	0.034 ± 0.000 i	nd	0.090 ± 0.000 e	nd	0.009 ± 0.000 fgh	nd
Pirata	nd	nd	nd	0.012 ± 0.000 j	0.080 ± 0.002 o	0.012 ± 0.000 l	nd	0.030 ± 0.001 l	nd	0.004 ± 0.000 ghi	nd
CB2011.280	nd	nd	nd	0.025 ± 0.000 d	0,318 ± 0.006 g	0.046 ± 0.000 e	nd	0.107 ± 0.002 d	nd	nd	nd
CB2011.273	nd	nd	nd	0.017 ± 0.000 h	0.214 ± 0.006 j	0.042 ± 0.001 f	nd	0.121 ± 0.003 c	nd	nd	nd
CB2011.098	nd	nd	nd	0.057 ± 0.000 b	0.343 ± 0.001 f	0.059 ± 0.000 d	0.071 ± 0.000 a	0.160 ± 0.001 b	nd	0.047 ± 0.001 d	nd
CB2011.253	nd	nd	nd	0.014 ± 0.000 i	0.205 ± 0.000 k	0.011 ± 0.000 g	0.009 ± 0.000 d	0.038 ± 0.000 e	nd	0.015 ± 0.000 f	nd
CB2012.076	nd	nd	nd	0.003 ± 0.000 l	0.019 ± 0.000 s	0.003 ± 0.000 n	0.004 ± 0.000 f	0.005 ± 0.000 n	nd	0.002 ± 0.002 hi	nd
CB2012.176	nd	nd	nd	Nd	0.237 ± 0.000 i	nd	nd	nd	nd	nd	nd
CB2012.347	nd	nd	nd	Nd	nd	nd	nd	nd	nd	nd	nd
CB2011.122	nd	nd	nd	Nd	0.145 ± 0.001 l	0.015 ± 0.000 k	0.011 ± 0.000 c	0.047 ± 0.000 i	nd	nd	nd
CB2011.375	nd	nd	nd	Nd	0.050 ± 0.000 q	0.009 ± 0.000 m	nd	0.027 ± 0.000 m	nd	nd	nd
CB2012.028	nd	nd	nd	Nd	0.021 ± 0.000 s	0.003 ± 0.000 n	0.003 ± 0.000 g	0.005 ± 0.000 n	nd	nd	nd
CB2012.063	nd	nd	nd	Nd	0.034 ± 0.000 r	0.004 ± 0.000 n	0.004 ± 0.000 e	0.007 ± 0.000 n	nd	nd	nd
CB2012.128	nd	nd	nd	Nd	0.007 ± 0.000 t	nd	nd	nd	nd	nd	nd
CB2012.208	nd	nd	nd	Nd	0.665 ± 0.001 b	0.058 ± 0.000 d	nd	0.179 ± 0.000 a	nd	nd	nd

CB2012.253	nd	nd	nd	Nd	0.584 ± 0.000 b	0.039 ± 0.000 l	nd	0.090 ± 0.000 k	nd	nd	nd
CB2012.350	nd	nd	nd	Nd	0.006 ± 0.000 t	nd	nd	nd	nd	nd	nd
CB2012.361	nd	nd	nd	Nd	0.019 ± 0.001 s	0.147 ± 0.004 b	nd	nd	0.156 ± 0.004 a	0.353 ± 0.014 a	nd
CR2012.363	nd	nd	nd	Nd	nd	nd	nd	nd	0.009 ± 0.000 c	nd	nd
CR2002.8	nd	nd	0.031 ± 0.000 a	Nd	nd	nd	nd	0.170 ± 0.000	nd	nd	0.018 ± 0.000 b
TR2012.078	0.057 ± 0,000 a	0.015 ± 0.000 a	nd	Nd	nd	0.170 ± 0.000 a	0.023 ± 0.001 b	nd	nd	nd	0.022 ± 0.000 a
TY2012.365	nd	nd	nd	0.002 ± 0.000 m	0.003 ± 0.000 tu	0.017 ± 0.000 jk	nd	nd	0.015 ± 0.000 b	0.033 ± 0.000 e	nd
CB2011.568	nd	nd	nd	Nd	nd	0.074 ± 0.000 c	nd	nd	nd	0.107 ± 0.000 c	nd
BWF	nd	nd	nd	Nd	nd	nd	nd	nd	nd	nd	nd
VR808	nd	nd	nd	Nd	nd	nd	nd	nd	nd	nd	nd

Table S3: Individual hydroxycinnamic acid derivatives concentrations (g kg^{-1} fresh weight) by HPLC-DAD in fresh samples of *Solanum tuberosum*. Where: 3CQA: 3-caffeoylquinic acid, 5CQA: 5-caffeoylquinic acid and 4CQA: 4-caffeoylquinic acid. Different letters in a column indicate the presence of statistically significant differences according the Tukey's multiple range test (P ≤ 0.05; n = 3).

Genotype	3CQA	5CQA	4CQA
CB2011.104	0.358 ± 0.012 a	1.964 ± 0.012 a	0.119 ± 0.001 b
CB2011.119	0.078 ± 0.000e	1.125 ± 0.000 ef	0.145 ± 0.000 b
CB2011.189	0.029 ± 0.000 kl	0.549 ± 0.003 k	0.069 ± 0.000 b
CB2011.247	0.023 ± 0.000 klmn	0.779 ± 0.000 hi	0.061 ± 0,000 b
CB2011.569	0.041 ± 0.000 j	0.729 ± 0.012 i	0.098 ± 0.001 b
CB2011.616	0.030 ± 0.000 k	0.789 ± 0.017 hi	0.084 ± 0.002 b
CB2012.096	0.089 ± 0.001 cd	1.204 ± 0.010 e	0.162 ± 0.002 b
CB2012.196	0.077 ± 0.000 e	0.644 ± 0.002 j	0,100 ± 0.000 b
CB2011.211	0.056 ± 0.000 gh	1.172 ± 0.006 e	0.116 ± 0.000 b
Pirata	0.046 ± 0.002 ij	0.289 ± 0.010 mn	0.060 ± 0.003 b
CB2011.280	0.027 ± 0.001 kl	0.837 ± 0.016 h	0.075 ± 0.001 b
CB2011.273	0.059 ± 0.002 g	1.390 ± 0.036 cd	0.133 ± 0.001 b
CB2011.098	0.018 ± 0.004 mno	0.525 ± 0.090 k	0.037 ± 0.009 b
CB2011.253	0.059 ± 0.000 ij	1.054 ± 0.001 f	0.582 ± 0.047 a
CB2012.076	0.025 ± 0.001 klm	0.237 ± 0.005 mno	0.036 ± 0.001 b
CB2012.176	0.012 ± 0.000 op	0.166 ± 0.006 pqr	0.017 ± 0.001 b
CB2012.347	0.006 ± 0.000 pq	0.099 ± 0.004 rst	0.021 ± 0.001 b
CB2011.122	0.083 ± 0.001 de	1.402 ± 0.001 c	0.143 ± 0.001 b
CB2011.375	0.040 ± 0.001 j	0.407 ± 0.001 l	0.054 ± 0.010 b
CB2012.028	0.028 ± 0.000 kl	0.314 ± 0.000 m	0.044 ± 0.000 b
CB2012.063	0.022 ± 0.000 lmn	0.237 ± 0.000 mno	0.037 ± 0.000 b
CB2012.128	0.018 ± 0.000 mno	0.147 ± 0.001 qrs	0.024 ± 0.000 b
CB2012.208	0.103 ± 0.001 b	1.492 ± 0.003 b	0.180 ± 0.001 b
CB2012.253	0.045 ± 0.001	1.533 ± 0.048 b	0.114 ± 0.003 b
CB2012.350	0.011 ± 0.000 opq	0.072 ± 0.000 st	0.013 ± 0.000 b
CB2012.361	0.053 ± 0.001 ghi	1.546 ± 0.044 b	0.126 ± 0.003 b
CR2012.363	0.015 ± 0.002 no	0.219 ± 0.006 nop	0.029 ± 0.001 b
CR2002.8	0.052 ± 0.000 ghi	0.966 ± 0.002 g	0.126 ± 0.000 b
TR2012.078	0.092 ± 0.006 c	1.311 ± 0.083 d	0.156 ± 0.010 b
TY2012.365	0.050 ± 0.000 hi	0.404 ± 0.001 l	0.076 ± 0.000 b
CB2011.568	0.068 ± 0.001 f	0.781 ± 0.004 hi	0.155 ± 0.043 b
BWF	0.005 ± 0.000 pq	0.108 ± 0.001 rst	0.013 ± 0.000 b
VR808	0.003 ± 0.000 q	0.055 ± 0.000 t	0.007 ± 0.000 b

Table S4: Individual hydroxycinnamic acid derivatives concentrations (g kg^{-1} fresh weight) by HPLC-DAD in cooked samples of *Solanum tuberosum*. Where: 3CQA: 3-caffeoylquinic acid, 5CQA: 5-caffeoylquinic acid and 4CQA: 4-caffeoylquinic acid. Different letters in a column indicate the presence of statistically significant differences according the Tukey's multiple range test (P ≤ 0.05; n = 3).

Genotype	3CQA	5CQA	4CQA
CB2011.104	0.230 ± 0.006 a	3.510 ± 0.331 a	0.489 ± 0.046 a
CB2011.119	0.195 ± 0.000 c	1.097 ± 0.002 bcd	0.305 ± 0.000 b
CB2011.189	0.169 ± 0.019 ef	0.563 ± 0.064 fghijk	0.229 ± 0.028 bcdefg
CB2011.247	0.077 ± 0.000 kl	0.492 ± 0.000 hijkl	0.133 ± 0.019 hijklmn
CB2011.569	0.057 ± 0.006 mn	0.444 ± 0.025 ijklm	0.092 ± 0.003 jklmno
CB2011.616	0.089 ± 0.003 ijk	0.444 ± 0.010 ijklm	0.137 ± 0.023 hijklm
CB2012.096	0.222 ± 0.000 ab	0.877 ± 0.002 cdef	0.263 ± 0.001 bc
CB2012.196	0.213 ± 0.002 b	0.847 ± 0.013 cdef	0.248 ± 0.001 bcde
CB2011.211	0.172 ± 0.000 ef	0.830 ± 0.000 cdefg	0.207 ± 0.002 cdefgh
Pirata	0.085 ± 0.000 ijk	0.204 ± 0.002 lm	0.081 ± 0.001 klmno
CB2011.280	0.110 ± 0.005 gh	0.779 ± 0.033 defgh	0.167 ± 0.007 efghijk
CB2011.273	0.224 ± 0.003 ab	1.050 ± 0.014 bcde	0.283 ± 0.004 bc
CB2011.098	0.188 ± 0.001 cd	0.864 ± 0.012 cdef	0.230 ± 0.002 bcdefg
CB2011.253	0.098 ± 0.000 hi	0.822 ± 0.002 cdefgh	0.159 ± 0.003 fghijkl
CB2012.076	0.066 ± 0.001 lm	0.227 ± 0.004 lm	0.079 ± 0.001 lmno
CB2012.176	0.187 ± 0.007 efghi	0.610 ± 0.030 fghij	0.201 ± 0.006 cdefghi
CB2012.347	0.046 ± 0.000 no	0.173 ± 0.000 lm	0.056 ± 0.000 mno
CB2011.122	0.234 ± 0.006 a	0.496 ± 0.048 ghijkl	0.142 ± 0.014 ghijklm
CB2011.375	0.094 ± 0.000 i	0.329 ± 0.000 jklm	0.107 ± 0.002 jklmno
CB2012.028	0.076 ± 0.000 kl	0.321 ± 0.000 jklm	0.090 ± 0.000 jklmno
CB2012.063	0.092 ± 0,001 ij	0.178 ± 0.003 jklm	0.087 ± 0.001 jklmno
CB2012.128	0,114 ± 0.002 g	0.331 ± 0.001 lm	0.113 ± 0.001 ijklmno
CB2012.208	0.178 ± 0.001 de	1.231 ± 0.007 b	0.257 ± 0.001 bcd
CB2012.253	0.098 ± 0.000 hi	0.822 ± 0.002 cdefgh	0.159 ± 0.003 fghijkl
CB2012.350	0.055 ± 0.000 mn	0.126 ± 0.000 m	0.055 ± 0.000 mno
CB2012.361	0.122 ± 0.001 g	0.738 ± 0.006 efghi	0.173 ± 0.002 defghi
CR2012.363	0.052 ± 0.000 n	0.304 ± 0.001 jklm	0.073 ± 0.001 lmno
CR2002.8	0.080 ± 0.000 jk	0.403 ± 0.002 jklm	0.107 ± 0.000 jklmno
TR2012.078	0.161 ± 0.001 f	1.118 ± 0.024 bc	0.240 ± 0.006 bcdef
TY2012.365	0.051 ± 0.001 n	0.237 ± 0.004 klm	0.063 ± 0.001 mno
CB2011.568	0.088 ± 0.000 ijk	0.319 ± 0.001 jklm	0.098 ± 0.001 jklmno
BWF	0.028 ± 0.000 p	0.111 ± 0.002 m	0.035 ± 0.001 o
VR808	0.034 ± 0.001 op	0.195 ± 0.000 lm	0.048 ± 0.000 no
